# Patient safety assurance in the age of defensive medicine: a review

**DOI:** 10.1186/s13037-022-00319-8

**Published:** 2022-02-17

**Authors:** Amrita Shenoy, Gopinath N. Shenoy, Gayatri G. Shenoy

**Affiliations:** 1 Assistant Professor of Healthcare Administration, University of Baltimore, College of Public Affairs, School of Health and Human Services, 1420 N. Charles Street, Baltimore, MD 21201 USA; 2 Medical Malpractice Attorney/Senior Medicolegal Consultant, Post-Graduate Examiner of Law (LLM & PhD) at the University of Mumbai, Former Honorary Professor of Obstetrics/Gynecology at K J Somaiya Medical College and Hospital, Former President and Post-Graduate Examiner of Obstetrics/Gynecology at the College of Physicians and Surgeons of Bombay, and Former Member of the Consumer Disputes Redressal Forum, Mumbai Suburban District, State Government of Maharashtra, Mumbai, India; 3 Former Assistant Professor and Diplomate of the National Board (DNB) Faculty of Anesthesiology, K J Somaiya Medical College and Hospital, Mumbai, Maharashtra India

**Keywords:** Defensive medicine, Quality of care, Medical errors, Taxonomy, Patient safety

## Abstract

The definition of defensive medicine has evolved over time given various permutations and combinations. The underlying meaning, however, has persisted in its relevance towards two classifications, positive and negative defensive medicine. Positive defensive medicine is specific to overutilization, excessive testing, over-diagnosing, and overtreatment. Negative defensive medicine, on the contrary, is specific to avoiding, referring, or transferring high risk patients. Given the above bifurcation, the present research analyzes defensive medicine in the landscape of medical errors. In its specificity to medical errors, we consider the cognitive taxonomies of medical errors contextual to execution and evaluation slips and mistakes. We, thereafter, illustrate how the above taxonomy interclasps with five classifications of medical errors. These classifications are those that involve medical errors of operative, drug-related, diagnostic, procedure-related, and other types. This analytical review illustrates the nodular frameworks of defensive medicine. As furtherance of our analysis, this review deciphers the above nodular interconnectedness to these error taxonomies in a cascading stepwise sequential manner. This paper was designed to elaborate and to stress repeatedly that practicing defensive medicine entails onerous implications to physicians, administrators, the healthcare system, and to patients. Practicing defensive medicine, thereby, is far from adhering to those optimal healthcare practices that support quality of care metrics/milestones, and patient safety measures. As an independent standalone concept, defensive medicine is observed to align with the taxonomies of medical errors based on this paper’s diagrammatic and analytical inference.

## Background

There are four principles of clinical or biomedical ethics [1]. These four principles, expounded in Beauchamp and Childress’ book titled, Principles of Biomedical Ethics, are enlisted as *beneficence, nonmaleficence, autonomy*, and *justice* [1, 2]. Physicians, in general, practice their medical specialties based on the above principles of biomedical ethics.

In medical practice, most of the times, clinical outcomes end as expected. There are other instances in which the outcome culminates into an unexpected consequence even when medicine was practiced, ethically, appropriately, and with all care and caution. After such an incidence, in all future cases, the physician contemplates upon a defensive medical practice and starts ordering an array of laboratory tests and referrals to reinforce his diagnosis.

This practice of comprehensively considering and ordering multiple laboratory tests, over-investigating, or over-utilizing available resources is referred to as ‘Defensive Medicine’. The definitions of Defensive Medicine have undergone many changes in the past.

In 1999, the United States (US) Congress defined Defensive Medicine in its document of the Office of Technology Assessment (OTA) as that occurrence when doctors order tests, procedures, or visits, or avoid high-risk patients or procedures, primarily (but not necessarily solely) to reduce their exposure to malpractice liability [3].

In 2000, Summerton defined defensive medicine as the ordering of treatments, tests, and procedures for the purpose of protecting the doctor from criticism rather than diagnosing or treating the patient [4].

In 2004, Toker and coauthors redefined defensive medicine as a physician’s deviation from what is considered to be good practice to prevent complaints from patients or their families [5].

In 2012, Sethi and coauthors reframed the above definitions as medical practices that may exonerate physicians from liability without significant benefit to patients, (and) can be categorized as either positive or negative [6].

In 2013, Ortashi and researchers recomposed its definition as a doctor’s deviation from usual behavior or that considered good practice, to reduce or prevent complaints or criticism by patients or their families [7].

Limiting exposure to malpractice liability, mitigating complaints from patients, or avoiding high risk patients are a few of the numerous granulated elements within the concept of defensive medicine [4–7]. In general, physicians tend to defensively practice medicine to proactively manage the undesired outcomes of malpractice lawsuits.

The underlying basis of defensive medicine is, therefore, to decipher that in a malpractice case, the physician has taken all care, caution, and safety measures to go above and beyond the accepted thresholds of clinical practice [8] and the expected standard of care.

Defensive medicine, therefore, becomes an unintended consequence of medical practice. Given the two extremes, defensive medicine is classified as positive defensive medicine or negative defensive medicine [6–9]. At one extreme, positive defensive medicine is observed when physicians provide too much care with excessive testing, overutilization of resources, multiple ordering, or referrals [6–9]. At the other extreme, negative defensive medicine is observed when physicians provide too little care by avoiding, referring, or transferring high risk patients [6–9]. Positive and negative defensive medicine affects and delays excessive healthcare spending and timely healthcare access, respectively [9–11].

Studdert and coauthors conducted an empirical study in which the binary outcome variable was reporting defensive medicine. Their study inferred that defensive medicine was highly prevalent among physicians that pay the most for liability insurance in the region of study. They, furthermore, inferred that defensive medicine has potentially serious implications for cost, access, and quality of care, both technical and interpersonal [12].

This paper aims to demonstrate how defensive medicine culminates into medical errors implying systemic risks to various healthcare stakeholders. Instrumental to this aim, we trifurcate our demonstration with three concepts: (1) defensive medicine’s nodular framework and the taxonomies of medical errors, (2) how defensive medicine and medical errors align in its elemental framework, and (3) consequences to healthcare stakeholders such as providers, patients, and administrators.

This study’s research questions burgeon into three objectives. First, we explain the alignment of the nodes of defensive medicine vis-à-vis the taxonomy of medical errors. Second, we discern how defensive medicine directly conforms to this taxonomic alignment with respect to medical errors in its elemental construction. In this process, we illustrate the above alignment of defensive medicine and medical errors in a cascading schematic flowchart. Third, we, additionally, explore defensive medicine within the landscape of its systemic risks and consequences contextual to physicians, administration, and patients.

The above research questions sequentially ligate three defensive medicine spectra to the taxonomy of medical errors. The objective of the first research question is to present that defensive medicine aligns and interconnects with the taxonomic categories of medical errors. The objective of the second research question is to interlink and transpire this framework into a visual depiction of defensive medicine’s alignment to medical error taxonomies. The objective of the third research question is to reasonably expound upon the risk implications of defensive medicine to various healthcare stakeholders.

### The framework of defensive medicine

**Fig. 1:**
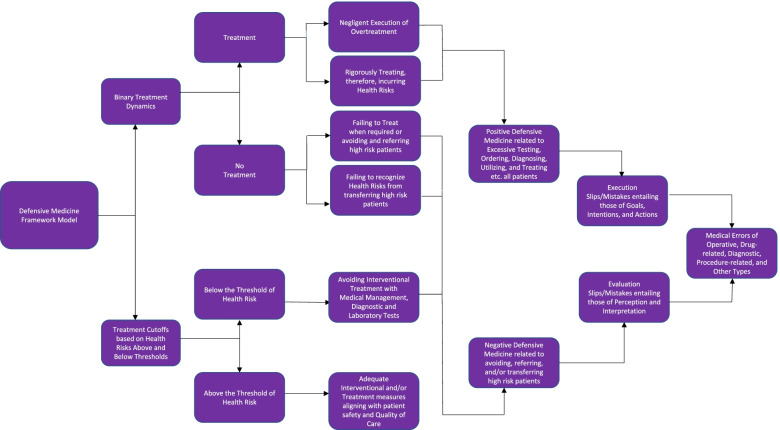
Cascading Algorithm representing Defensive Medicine framework model’s interconnection to the Taxonomies of Medical Errors. [Sources: Source(s) of: (1) The Nodes of the Defensive Medicine framework model: (i) Frakes MD. The surprising relevance of medical malpractice law. U. Chi. L. Rev. 2015;82(1):317–391. Available from: https://www.jstor.org/stable/43234698, and (2) Medical Error Taxonomies: (i) Zhang J, Patel VL, Johnson TR, Shortliffe EH. A cognitive taxonomy of medical errors. J Biomed Inform. 2004;37(3):193–204. 10.1016/j.jbi.2004.04.004, and (ii) Andel C, Davidow SL, Hollander M, Moreno DA. The economics of health care quality and medical errors. J Health Care Finance. 2012;39(1):39–50. Available from: https://pubmed.ncbi.nlm.nih.gov/23155743/]

Figure 1 visually and schematically represents the cascading flowchart in a stepwise sequential manner. This flowchart interconnects defensive medicine nodes and networks as part of this analysis. In essence, the above defensive medicine framework, its positive and negative defensive medicine subtypes, interrelation to execution and evaluation slips/mistakes are observed to culminate into five types of medical errors.

The mechanics of the defensive medicine framework, as conceptualized by Michael Frakes, consists of ‘Binary Treatment Dynamics’ and ‘Threshold Cutoffs’ [13]. The binary treatment dynamics bifurcates into ‘Treatment’ and ‘No Treatment’ nodes [13]. The first dynamic of treatment entails two aspects, first, negligent execution of treatment, and second, overtreatment [13]. Overtreating the sick and ailing is believed to further exacerbate their compromised health statuses, and is, therefore, not advisable [13]. The second dynamic of no treatment also entails two aspects, first, failing to treat when required, and second, failing to recognize health risks from not treating high risk patients [13].

The second node of this defensive medicine framework, essentially Threshold Cutoffs, is specific to health risks [13]. Physicians, in general, recommend adequate interventional treatment if the cutoff threshold is above the health risk [13]. Physicians, conversely, are more inclined towards non-interventional treatment, and thus, manage a sickness and its symptoms with medications if the cutoff threshold is below the health risk [13]. In the latter case, diagnostic procedures such as X-Rays, Magnetic Resonance Imaging (MRI), and Computed Tomography (CT) scans as well as laboratory tests become more applicable.

In reference to Fig. 1, on the one hand, the dynamics of treatment, may signify positive defensive medicine in relation to overutilization of healthcare services, excessive testing, superfluous ordering of tests, over diagnosing, and over treating [6, 7]. On the other hand, the dynamics of no treatment may signify negative defensive medicine in its relation to avoiding, referring, and/or transferring high risk patients [6, 7]. Adequate interventional and/or treatment measures that are, therefore, in alignment with the patient’s healthcare needs conform to quality of care and safety measures.

Medical errors, as conceptualized by Zhang et al., are categorized as the cognitive taxonomy of medical errors [14]. This taxonomy has medical errors bifurcated into slips and mistakes [14]. These are further sub-categorized into execution and evaluation slips/mistakes [14]. Execution slips/mistakes are granulated into those oriented towards goals, intentions, action specific−/execution- ones [14]. Evaluation slips/mistakes are granulated into those that are oriented towards perception and interpretation ones [14].

Four types of medical errors, as delineated by Leape et al., are *diagnostic, treatment, preventive, and other errors* [15]. The Institute of Medicine (IOM) outlined strategies for improvement and spotlighted progress for curbing those errors in its *To Err Is Human* report in November 1999 [16]. Five types of medical errors, as classified by Andel et al., are *operative*, *drug-related*, *diagnostic*, *therapeutic*, *procedure-related*, and *other* [17].

### The risks of defensive medicine

The risk of defensive medicine pervades not only to the physician, but also to the patient, hospital administration, and the system. In general, risks to the physician are inclusive but not limited to an increase in accountability for excessively ordering a gamut of tests, treatment lines, or procedures. The above implicates an increase in the likelihood to be potentially sued in case of *missed*, *delayed*, *wrong*, or *overdiagnosis*. As a foreseeable consequence, a record of malpractice litigation increases the physician’s professional indemnity insurance premiums.

Defensive medicine, additionally, has administrative implications from an operational perspective. Physicians engaging in defensive medicine increase and augment tests and orders for ailments that may be remotely related to the actual diagnosis. Excessive laboratory testing implicates the increased need for well-staffed human resources personnel that are qualified to perform those tests to provide reports and results.

In short-staffed situations, the hospital may need to employ additional personnel to furnish tests in a timely manner, thereby, stressing increased hospital resources and operations. Excessive hospitalizations or procedures may divert essential resources potentially engaging the need to better utilize existing resources.

Defensive medicine imposes systemic risks from healthcare overconsumption and financial viewpoints. Excessive utilization and testing, financially stresses an already overstretched healthcare system whose costs run dominantly in trillions of dollars. Excessive testing potentially increases healthcare wastes owing to excessive processing, overutilization, and overproduction.

The patient, nevertheless, is not exempt from the risks arising from defensive medicine. The patient is also a systemic stakeholder, and one that bears the financial consequences of excessive ordering and testing, in circumstances of self-payment or self-insurance. The onus of paying for excessive testing is partly transferred to public or private healthcare insurance companies, in the event the patient is insured. Defensive medicine is primarily prevalent in Obstetrics/Gynecology cases of caesarean sections and in Radiology [10, 18, 19].

This paper, first, qualitatively examines its research questions which involves defensive medicine framework nodes and medical errors classifications. Second, it describes the interconnection of defensive medicine in alignment to five types of medical errors notwithstanding new classification types that may develop in the future. Third, this paper’s schematic flowchart, that sequentially depicts an alignment network, is limited in its nature to only those specific framework nodes and processes.

There are some strengths of this paper and its functions. First, this paper makes it feasible to visualize the construction and alignment of defensive medicine’s framework to the cognitive taxonomy of medical errors occurring in its elemental form. Second, this visual depiction may facilitate analysts and theorists to further develop the interconnections of this network. Third, it may better equip readers to extend this research into applying this nodular alignment network to more medical malpractice laws such as Respondeat Superior, Res Ipsa Loquitur, Informed Consent, Expert Witness Testimony, and Patient Safety.

Acquiring data on each taxonomy of the above medical errors from either a survey or data repository may be useful in incorporating a quantitative aspect to this research. Second, it would be meaningful to update this research with the new taxonomies that evolve in the future, especially, those relevant to additional medical error categories. Third, it would be exceedingly panoramic to construct an inter-aligning network concurrently overlapping with malpractice themes such as Respondeat Superior [20], Res Ipsa Loquitur, Expert Witness Testimony, Informed Consent [21], and Patient Safety [22].

## Conclusion

The purpose of this analytical narrative research was to analyze defensive medicine through the lenses of its nodes, sub-nodes, and granulated components.

We analyzed the alignment of defensive medicine within the scope of its framework, positive and negative sub-types, and the taxonomies of medical errors. We, in this process, depicted a sequential stepwise schematic flowchart.

This flowchart visualized the alignment of defensive medicine and its components to execution and evaluation slips and mistakes. These slips and mistakes, thereafter, inherently formed components of medical errors. We, thereby, connected defensive medicine to the taxonomies of medical errors.

Defensive medicine, thereby, entails onerous implications to physicians, administrators, the healthcare system, and to patients. Practicing defensive medicine, therefore, is far from adhering to those optimal healthcare practices that support quality of care metrics, milestones, and measures.

The overarching goal of this analytical review was to realize that defensive medicine interclasps with the taxonomies of medical errors. Defensive medicine, as an independent standalone concept is, therefore, observed to be qualitatively and visually far from aligning with patient safety milestones, measures, and metrics.

## Data Availability

Not applicable.

## Abbreviations

CT: Computed Tomography
IOM: Institute of Medicine
MRI: Magnetic Resonance Imaging
OTA: Office of Technology Assessment

## References

[1] Beauchamp TL, Childress JF (2001). Principles of biomedical ethics. 5th.

[2] Varkey B (2021). Principles of Clinical Ethics and Their Application to Practice. Med Princ Pract.

[3] Defensive medicine: definition and causes. In: Defensive medicine and medical malpractice. Washington (DC): U.S. Congress, Office of Technology Assessment Government Printing Office. 1994. p. 21–37. Available from: https://ota.fas.org/reports/9405.pdf

[4] Summerton N (2000). Trends in negative defensive medicine within general practice. Br J Gen Pract.

[5] Toker A, Shvarts S, Perry ZH, Doron Y, Reuveni H (2004). Clinical guidelines, defensive medicine, and the physician between the two. Am J Otolaryngol.

[6] Sethi MK, Obremskey WT, Natividad H, Mir HR, Jahangir AA (2012). Incidence and costs of defensive medicine among orthopedic surgeons in the United States: a national survey study. Am J Orthop.

[7] Ortashi O, Virdee J, Hassan R, Mutrynowski T, Abu-Zidan F (2013). The practice of defensive medicine among hospital doctors in the United Kingdom. BMC Med Ethics.

[8] Baungaard N, Skovvang P, Assing Hvidt E, Gerbild H, Kirstine Andersen M, Lykkegaard J (2020). How defensive medicine is defined and understood in European medical literature: protocol for a systematic review. BMJ Open.

[9] Black L (2007). Effects of Malpractice Law on the Practice of Medicine. AMA J Ethics.

[10] Frakes M, Gruber J (2020). Defensive medicine and obstetric practices: evidence from the military health system. J Empir Leg Stud.

[11] Hermer LD, Brody H (2010). Defensive medicine, cost containment, and reform. J Gen Intern Med.

[12] Studdert DM, Mello MM, Sage WM, DesRoches CM, Peugh J, Zapert K (2005). Defensive medicine among high-risk specialist physicians in a volatile malpractice environment. JAMA..

[13] Frakes MD (2015). The surprising relevance of medical malpractice law. U Chi L Rev.

[14] Zhang J, Patel VL, Johnson TR, Shortliffe EH (2004). A cognitive taxonomy of medical errors. J Biomed Inform.

[15] Leape L, Lawthers AG, Brennan TA, Johnson WG (1993). Preventing Medical Injury. Qual Rev Bull.

[16] Institute of Medicine (US) Committee on Quality of Health Care in America. To Err is Human: Building a Safer Health System. In: Kohn LT, Corrigan JM, Donaldson MS, editors. Washington (DC): National Academies Press (US); 1999. PMID: 25077248. Available from: https://www.nap.edu/resource/9728/To-Err-is-Human-1999%2D%2Dreport-brief.pdf. 25077248

[17] Andel C, Davidow SL, Hollander M, Moreno DA (2012). The economics of health care quality and medical errors. J Health Care Finance.

[18] Zwecker P, Azoulay L, Abenhaim HA (2011). Effect of fear of litigation on obstetric care: a nationwide analysis on obstetric practice. Am J Perinatol.

[19] Ramella S, Mandoliti G, Trodella L, D’Angelillo RM (2015). The first survey on defensive medicine in radiation oncology. Radiol Med.

[20] Shenoy A, Shenoy GN, Shenoy GG (2021). Respondeat superior in medicine and public health practice: the question is–who is accountable for whom?. Ethics Med Public Health.

[21] Shenoy A, Shenoy GN, Shenoy GG. Informed consent: Legalities, perspectives of physicians and patients, and practices in OECD/non-OECD countries. Méd Palliative. 2021. In press. 10.1016/j.medpal.2021.07.004.

[22] Shenoy A (2021). Patient safety from the perspective of quality management frameworks: a review. Patient Saf Surg.

